# Identification of a novel transforming growth factor-β (TGF-β6) gene in fish: regulation in skeletal muscle by nutritional state

**DOI:** 10.1186/1471-2199-11-37

**Published:** 2010-05-12

**Authors:** Bruria Funkenstein, Elena Olekh, Sonia B Jakowlew

**Affiliations:** 1 National Institute of Oceanography, Israel Oceanographic and Limnological Research, Tel Shikmona, P.O.B 8030, Haifa 31080, Israel; 2 National Cancer Institute, Center for Cancer Training, Cancer Training Branch, Bethesda, Maryland 20892-8346, USA

## Abstract

**Background:**

The transforming growth factor-β (TGF-β) family constitutes of dimeric proteins that regulate the growth, differentiation and metabolism of many cell types, including that of skeletal muscle in mammals. The potential role of TGF-βs in fish muscle growth is not known.

**Results:**

Here we report the molecular characterization, developmental and tissue expression and regulation by nutritional state of a novel TGF-β gene from a marine fish, the gilthead sea bream *Sparus aurata*. *S. aurata *TGF-β6 is encoded by seven exons 361, 164, 133, 111, 181, 154, and 156 bp in length and is translated into a 420-amino acid peptide. The exons are separated by six introns: >643, 415, 93, 1250, 425 and >287 bp in length. Although the gene organization is most similar to mouse and chicken TGF-β2, the deduced amino acid sequence represents a novel TGF-β that is unique to fish that we have named TGF-β6. The molecule has conserved putative functional residues, including a cleavage motif (RXXR) and nine cysteine residues that are characteristic of TGF-β. Semi-quantitative analysis of TGF-β6 expression revealed differential expression in various tissues of adult fish with high levels in skin and muscle, very low levels in liver, and moderate levels in other tissues including brain, eye and pituitary. TGF-β6 is expressed in larvae on day of hatching and increases as development progresses. A fasting period of five days of juvenile fish resulted in increased levels of TGF-β6 expression in white skeletal muscle compared to that in fed fish, which was slightly attenuated by one injection of growth hormone.

**Conclusion:**

Our findings provide valuable insights about genomic information and nutritional regulation of TGF-β6 which will aid the further investigation of the *S. aurata *TGF-β6 gene in association with muscle growth. The finding of a novel TGF-β6 molecule, unique to fish, will contribute to the understanding of the evolution of the TGF-β family of cytokines in vertebrates.

## Background

The transforming growth factor-β (TGF-β) superfamily constitutes of a large number of structurally related, extracellular polypeptide growth factors that regulate a diverse spectrum of biological processes [1]. The TGF-β superfamily consists of over 50 structurally related ligands, many of which are categorized into three major subfamilies: TGF-β, bone morphogenetic protein (BMP) and activin/inhibin. Three TGF-β isoforms are known in mammals (TGF-β1, -β2, -β3) [2-7] and in birds (TGF-β2, -β3 and -β4) [8-11], and two in amphibians (TGF-β2, TGF-β5) [12,13].

Preliminary results from our laboratory showed expression of a TGF-β-like transcript in a marine fish, the gilthead sea bream *Sparus aurata *during early larval development [14,15]. The partial cloned fragment showed high similarity to chicken and mammalian TGF-β3. In recent years, evidence has accumulated suggesting the presence of at least three types of TGF-β in fish. Based on homologies with mammalian TGF-βs, it was suggested that TGF-β1 is present in rainbow trout, goldfish, carp, hybrid striped bass, plaice, gilthead sea bream and zebrafish [16-23]. TGF-β2 has been found in the carp, zebrafish and plaice [20,24,25]. TGF-β3 (partial sequence) was identified in Siberian sturgeon, rainbow trout, European eel and plaice [20,26] and in zebrafish [27]. The identification of fish TGF-β2 is somewhat confusing. First, two Genbank entries appeared for zebrafish TGF-β2, having 72% identity in the amino acid sequence of the mature TGF-β. Second, carp mature TGF-β2 is reported to be highly similar (93% identity) to human TGF-β2 [24].

TGF-β isoforms have overlapping biological actions and play critical roles during development, soft tissue repair, bone remodeling, inflammation and carcinogenesis. These isoforms are widely expressed and play a role during amphibian, avian and mammalian development [12,28,29]. Skeletal muscle regeneration and development are influenced by signal transduction pathways initiated by growth factors such as TGF-β, insulin-like growth factors (IGFs), and fibroblast growth factors. Major advance had been made in understanding the role of TGF-β and its closely related family member, myostatin (MSTN), in skeletal muscle ontogeny and postnatal physiology [reviewed in [30]]. Skeletal muscle express all three mammalian TGF-β isoforms (-β1, -β2 and -β3) [31,32]. *In vitro*, TGF-β1 mRNA is reduced while TGF-β2 and TGF-β3 are increased after differentiation (fusion) of the myoblast cell line C2C12 [31]. Others have shown that TGF-β inhibits muscle differentiation through functional repression of myogenic transcription factors by the TGF-β intracellular effector Smad3 [33]. Recently, the effects of TGF-β1, -β2 and -β3 on proliferation and differentiation of skeletal muscle myoblasts were compared using the C2C12 cell line and it was shown that all three TGF-β isoforms delay myoblast differentiation while increasing cellular proliferation [34]. TGF-β1 has been also implicated in muscle fibrosis following muscle injury [[35] and references therein].

Research on TGF-β in fish has been limited to its involvement in the immune system [23,36-38] and in reproduction [21,39,40], but no information is available to date (to our knowledge) on the possible involvement of TGF-β in fish muscle growth, which in fish that attain large body size is unique. In contrast to mammals and birds, fish skeletal muscles grow significantly after embryogenesis in post-larval life through continuous hyperplasia and hypertrophy (giving rise to the characteristic 'mosaic' of small- and large-diameter fibres in the white muscle), contributing to their large adult size [41].

To identify growth factors regulating muscle growth in fish of agricultural value, we report here on the cloning of a novel form of TGF-β from the gilthead sea bream *Sparus aurata *that we named TGF-β6 (following TGF-β4 and -β5 in chicken and *Xenopus*, respectively), which is expressed also in zebrafish, *Tetraodon, Takifugu*, medaka and stickleback. This TGF-β is expressed in various tissues including muscle and undergoes induction in white skeletal muscle following starvation, in a similar way to another member of the TGF-β superfamily, MSTN.

## Methods

### Materials

Oligonucleotides were prepared by Sigma (Rehovot, Israel) and The Midland Certified Reagent Company (Midland, TX). Restriction and modifying enzymes were purchased from Gibco BRL (Gaithersburg, MD, USA), New England Biolabs (Beverly, MA, USA) and Promega (Madison, WI, USA). T3, T7 and SP6 RNA polymerase primers were purchased from Promega and from Sigma. Radionucleotides were obtained from Amersham (Little Chalfont, UK) and from Dupont NEN (Boston, MA, USA).

### Fish and Tissues

Staged larvae and embryos of *S. aurata *were obtained from the National Center of Mariculture (Eilat, Israel) and from The Salt Company, Atlit. Juvenile fish were obtained from Kibbutz Ma'agan Michael, The Salt Company, Atlit and Mevo'ot-Yam School, Michmoret and kept in 700-L tanks at IOLR, Haifa at ambient temperature (20-25°C) in flow-through seawater and fed ad libitum. Embryos, larvae and tissues removed from decapitated fish were snap-frozen on dry ice and kept at -70°C until RNA extraction.

For evaluating the effect of nutrition and growth hormone (GH) on gene expression, juvenile fish (mean body weight 23 gr) were divided into 3 groups: (i) fasted for 5 days and then injected with PBS; (ii) fasted for 5 days and then injected with recombinant *S. aurata *GH (saGH, 1 μg/gr body weight); (iii) fed 2% of their body weight. All fish were sacrificed six hours after GH injection and white muscle was removed and snap-frozen for RNA extraction.

### RNA isolation, RT-PCR and Northern blot

Total RNA was extracted from different tissues of a single fish or pools of whole bodies of larvae and embryos by the guanidinium thiocyanate-cesium chloride gradient method, guanidinium-rapid method or by using TriReagent (Molecular Research Center, Cincinnati, OH), depending on the experiment. Poly(A^+^) RNA was obtained by affinity chromatography on oligo(dT)-cellulose columns (5 prime→3 prime, Inc, Boulder, CO).

RNA was separated by electrophoresis on 1% agarose gels, transferred to nylon membrane and hybridized essentially as described earlier [42]. Total RNA (10 μg) or poly(A^+^)RNA (1.2 μg) were reverse-transcribed into cDNA using oligo(dT)_12-18 _primer (500 ng) (Invitrogen, Paisley, UK), oligo(dT)_17_-adaptor primer (1 μg) or random hexamers (200 ng), depending on the experiment, and 200 units of Superscript II Reverse Transcriptase (Invitrogen).

**Figure 1:**
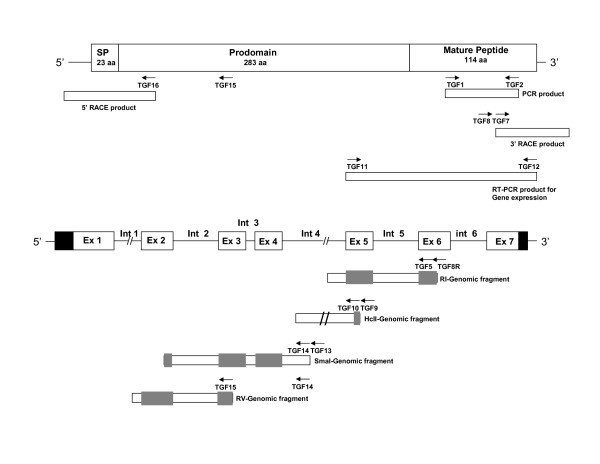
**Schematic structure and cloning strategies of TGF-β6**. TGF-β6 precursor contains a signal peptide (SP), an N-terminal peptide (prodomain) and a C-terminal mature peptide. Partial sea bream TGF-β6 cDNA sequence was initially obtained by using a pair of degenerate primers TGF1 and TGF2. The 3' end was obtained by 3'RACE using primers TGF7 and TGF8. Several genomic clones, containing exons 2-5 and part of exon 6, were obtained as described in detail in the Methods section. The 5' end was obtained by 5' RACE using primers TGF15 and TGF16. For clarity the MSTN gene and deduced protein have not been represented in their real proportions. Primers sequences are presented in Additional file 1.

Aliquots of 400-500 ng cDNA were used for PCR amplification of TGF-β using 2.5 U of Taq Polymerase (Sigma or New England Biolabs) and 25 pmol each of TGF-11 and TGF-12 primers (list of primers and their sequences are detailed in Additional file 1), which amplify a fragment of 489 bp. To verify that the amplified fragment in the expression studies is indeed the novel cDNA cloned in this study, fragments amplified from 7-day larval RNA and heart RNA were cloned and sequenced. An identity was found between the sequences of the cloned PCR fragments and the combined sequence obtained by 3'RACE and RI genomic fragment (see scheme in Fig. 1 for primers position and description of gene cloning below), thus confirming the identity of the amplified fragment as being novel TGF-β. Amounts of RNA in the RT-PCR reactions were controlled by amplification of β-actin also, as described earlier [43]. PCR products were analyzed on 1-2% agarose gels, stained with ethidium bromide and photographed under UV illumination. Conditions for PCR were optimized by using different amounts of cDNA and different number of cycles to ensure linearity. In some experiments transcripts of growth and differentiation factor-11 (GDF-11) and myostatin-1 (MSTN-1) were amplified as well using gene-specific primers (see Additional file 1).

### Densitometric analysis

Quantification of TGF-β, GDF-11, MSTN-1 and β-actin gene expression was performed using LISCAP (1995) Capture Application, version 1.0 and TINA version 2.07d program for densitometric analysis. Significant differences were determined by Tukey test after ANOVA using SPSS version 12.02 for Windows and Microsoft Excel 2000.

### Cloning of *S. aurata *TGF-β cDNA and gene

Cloning of full-length *S. aurata *novel TGF-β (TGF-β6) cDNA was performed using several steps as depicted in Fig. 1. Initially, a 225 bp PCR fragment of TGF-β was obtained by amplification of reverse-transcribed poly(A^+^)RNA (1.2 μg) extracted from larvae aged 7 days post-hatching [42,44] and degenerate oligomers designed on the basis of a conserved region of chicken TGF-β3 [8], spanning amino acids 330-335 (TGF-1) and amino acids 399-404 (TGF-2). The sequences of all primers are detailed in Additional file 1. Sequence analysis of the amplified fragment revealed high similarity to chicken TGF-β3. Based on the sequence obtained, several gene-specific primers were synthesized as detailed in Additional file 1.

The 3' end of the cDNA was obtained using FirstChoice RLM-RACE kit (Applied Biosystems/Ambion, Austin, TX, USA) according to the manufacturer instructions using total RNA (1.3 μg) from 7-day larvae reverse-transcribed using 3'RACE-adapter (Additional file 1). Amplification by PCR was performed by two successive reactions, using primers 3'RACE outer (Ambion) and gene-specific TGF-8 and followed by amplification using 3'RACE inner primer (Ambion) and the gene-specific primer TGF-7 (see scheme in Fig. 1). A fragment of about 400 bp was amplified, purified and cloned in pGEM-Teasy. BLAST analysis of the deduced amino acid sequence of the 3' end fragment revealed high homology with two GenBank entries (accession number XM_683088 and NM-194385), both annotated as zebrafish TGF-β2.

Since repeated attempts to clone the 5' end of the cDNA by 5' RACE method failed, most of the sequence was obtained by amplification of genomic DNA, essentially as described recently for cloning *S. aurata *MLC2 promoter by a linker-mediated PCR method [45]. Genomic DNA that was digested by several restriction enzymes: BamHI, EcoRI, EcoRV, HindIII, PstI, SacI, SmaI, HincII, PvuII, SspI and HpaI and was modified by T4 DNA polymerase to generate blunt ends, when needed. Following ligation with a linker DNA (oligo1 and oligo2), nested PCR was performed by gene walking using two linker-specific primers (L1 and L2) and several pairs of gene-specific primers, complementary to the 5' end of the already cloned *S. aurata *TGF-β. These pairs were: TGF-8R and TGF-5 to generate an amplified fragment from DNA digested with EcoRI, TGF-9 and TGF-10 to generate an amplified fragment from DNA digested with HincII, TGF-13 and TGF-14 to generate a fragment from DNA digested with SmaI, TGF-14 and TGF-15 to generate an amplified fragment of DNA digested with EcoRV (schematically illustrated in Fig. 1). The amplified genomic PCR products were separated by agarose gel electrophoresis. Fragments (as detailed above) in each round were gel-purified, cloned in pGEM-Teasy and sequenced. Over-lapping between the clones was verified by Clustal analysis.

The 5' end of the cDNA, including the signal peptide and 5' untranslated region were ultimately cloned by using 5' RLM-RACE Protocol of The FirstChoice RLM-RACE kit (Ambion) as recommended by the manufacturer, using total RNA (10 μg) from *S. aurata *7-day larvae, 5'RACE adaptor (Ambion) and random decamers for reverse transcription. Amplification by PCR was performed by two successive reactions, using primers 5'RACE outer (Ambion) and gene-specific TGF-15 followed by a second PCR using primers 5'RACE inner (Ambion) and the gene-specific TGF-16 (see Fig. 1). The 1200 bp amplified fragment was gel-purified, cloned in pGEM-Teasy and sequenced.

### Sequence analysis

Similarity searches of the sequenced DNA fragments and deduced amino acid sequence were done by BLASTN and BLASTX using nr/nt database of NCBI http://blast.ncbi.nlm.nih.gov/Blast.cgi[46]. A multiple-sequence alignment was performed using ClustalX version 2.0.8 [47]. A neighbor-joining (NJ) phylogenetic tree was constructed using Clustal X version 2.0.8 and MEGA version 4.0 [48]. Signal peptide length was predicted using SignalP 3.0 Server http://www.cbs.dtu.dk/services/SignalP/. N-glycosylation sites were predicted using NetNGlyc 1.0 Server http://www.cbs.dtu.dk/services/NetNGlyc/. Chromosomal localization of TGF-β genes and synteny analysis were performed using Ensembl release 56 http://www.ensembl.org/index.html[49].

## Results

### Characterization of *S. aurata *TGF-β cDNA and gene

**Figure 2:**
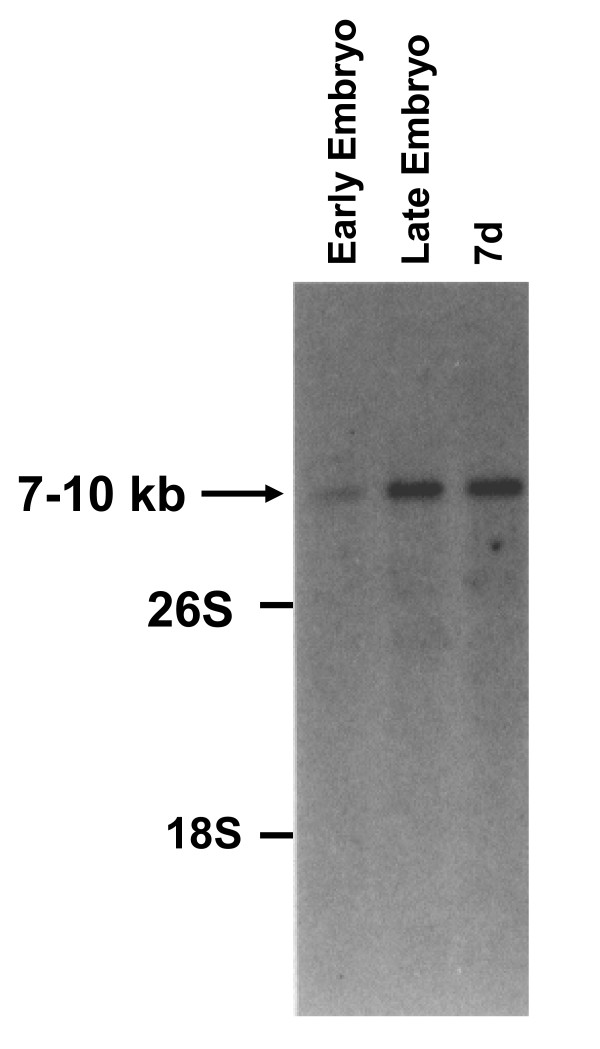
**Northern blot analysis of fish RNA hybridized with mouse TGF-β3 cDNA**. Poly(A^+^)RNA (2.5 μg) extracted from *S. aurata *at different developmental stages (early embryo, blastula/gastrula stage; late embryos, shortly before hatching; 7d, larvae collected 7 days after hatching) was hybridized to mouse TGF-β3 cDNA as described in Methods. Ethidium bromide staining shows 18S and 26S rRNA as a loading control.

Studies in mouse and chicken demonstrated TGF-β1, -β2, -β3 and -β4 expression in early developmental stages; hence, poly(A^+^)RNA isolated from 7-day larvae of *S. aurata *was hybridized to mammalian TGF-β1, TGF-β2 and TGF-β3 cDNAs. Only mouse TGF-β3 detected a transcript with an estimated size of 7-10 kb (data not shown). In order to determine the most suitable developmental stage for cloning TGF-β, poly(A^+^)RNA prepared from three different developmental stages (early embryos, late embryos and 7-day larvae) was hybridized with mouse TGF-β3 cDNA [50]. A transcript of the estimated size of 7-10 kb could be detected in early and late embryos as well as in 7-day larvae (Fig. 2). The levels of this mRNA transcript increased as development of the fish progressed.

PCR with degenerate TGF-β primers yielded a fragment of the predicted size (225 bp) using RNA from 7-day larvae. A BLAST analysis following cloning and sequencing of this fragment revealed strong homology with several vertebrate TGF-βs. The highest homology was found with chicken TGF-β3 (76%). This sequence was used to design primers in order to clone full-length TGF-β cDNA. The strategy for cloning *S. aurata *TGF-β sequences is presented schematically in Fig. 1 and described in more detail in the Methods section. The ends of the cDNA were obtained by 3' RACE and 5' RACE of RNA from 7-day larvae (Fig. 1).

**Figure 3:**
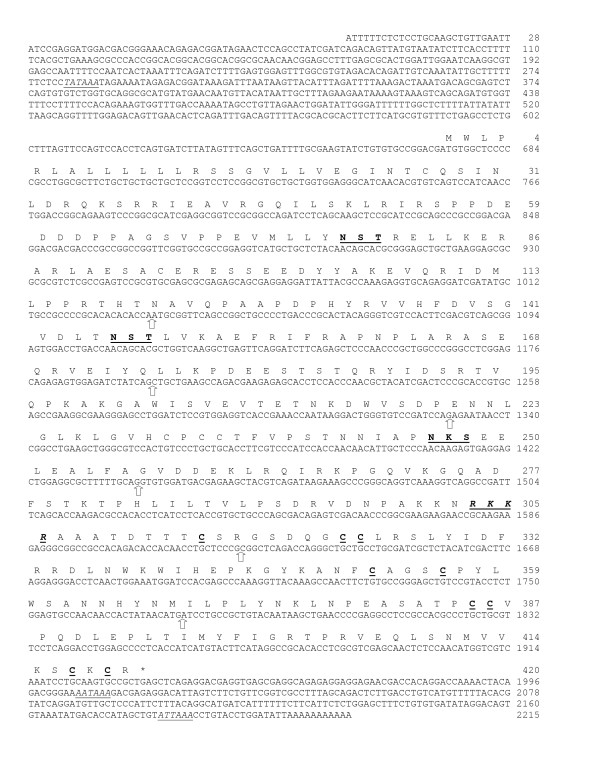
**Sequence analysis of *S. aurata *TGF-β6**. Nucleotide sequence of full length cDNA and deduced amino acid sequence, shown in single-letter code. Putative proteolytic site is in bold letters, underlined and italicized. The conserved nine cysteines in the mature peptide and three potential N-glycosylation sites in the prodomain are bold and underlined. The *asterisk *indicates the stop codon. Sites of intron insertions are indicated by empty thick arrows. TATAAA (TATA box motif) and polyadenylation signal motifs, AATAAA and ATTAAA, are underlined and italicized. Accession number for the cDNA sequence is FJ966093.

**Figure 4:**
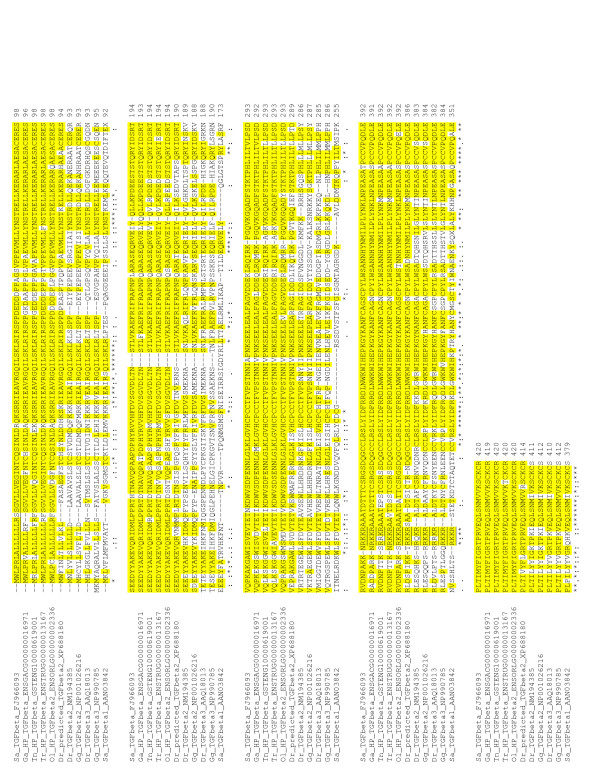
**Clustal analysis of TGF-β**. Clustal analysis of *S. aurata *TGF-β6 and several TGF-βs from fish and chicken. The predicted signal peptide is underlined. Identical residues to *S. aurata *TGF-β6 sequence are highlighted by a yellow shade. Proteolytic cleavage motifs are underlined.

The cDNA sequence reported herein and presented in Fig. 3 had been submitted to GenBank database (Accession No. FJ966093). The cDNA is 2215 bp long and contains an open reading frame of 1260 bp, with a deduced 420 amino acid long preproTGF-β. The signal sequence for secretion is predicted to be 23 residues; the prodomain is predicted to be 283 aa long and the carboxy-terminal mature TGF-β is 114 amino acid residues long as illustrated schematically in Fig. 1. The prepropeptide contains an RKKR proteolytic processing site in accordance with other members of the superfamily. The prodomain region of TGF-β precursor contains three potential N-linked glycosylation sites (N-X-T/S), similar to the two zebrafish TGF-β2, but is in contrast to *S. aurata *TGF-β1, which has four such sites. The mature TGF-β contains 7 cysteines common to other TGF-β superfamily members; however, like other TGF-βs, activins, GDF-8 (MSTN) and GDF-11, TGF-β6 contains one extra pair of cysteine residues (Fig. 3). Additionally, *S. aurata *TGF-β6 predicted mature peptide also displays the characteristic cysteine knot signature (CXCX-STOP) found in other species, as shown by the presence of a STOP codon at the second position after the sixth knot-forming cysteine. The first cysteine of the mature peptide appears at position 9, as in *Tetraodon nigroviridis*, *Takifugu rubripes*, medaka (*Oryzias latipes*) and stickleback (*Gasterosteus aculeatus*) but differs from other known fish TGF-β sequences in which the first cysteine appears in position 7 or 8 (Fig. 4). Within the 3' UTR, two potential polyadenylation signals are found: a distal one AATAAA and a second site, ATTAAA, located 16 bp upstream of a short polyadenylation sequence. The 5' untranslated region obtained by 5'RACE was found to be 672 bp long and this is predicted to be the transcription start site.

Alignment of the predicted mature bioactive peptide of the new *S. aurata *TGF-β revealed a very high homology of 93%, 93.9%, 96.5% and 98% amino acid identity with annotated sequences from *Fugu, Tetraodon*, medaka and stickleback, respectively. An identity of 86.8% was found with one entry of zebrafish TGF-β2 but a lower (69.3%) was found with a second zebrafish TGF-β2 entry (Fig. 4 and Table 1). Level of amino acid sequence identity with mammalian and chicken TGF-β2 and TGF-β3 mature peptide was similar (67.5-69.3%) (Table 1 and Fig. 4). In contrast, a very low identity was seen upon comparison with *S. aurata *TGF-β1 (45.6%) and zebrafish TGF-β3 (47.4%), supporting our conclusion that this TGF-β represents a novel TGF-β which was cloned also from zebrafish (Accession number XM_683088), but mistakenly was identified as TGF-β2. We called this novel peptide TGF-β6 because it is not similar to TGF-β1, 2 or 3 from fish. Multi-sequence alignment by CLUSTAL of deduced amino acid sequences of precursor TGF-β from *S. aurata*, the two zebrafish TGF-β2 sequences and predicted novel TGF-β from *Tetraodon, Fugu*, medaka and stickleback is presented in Fig. 4 and Table 2. A high homology with these four sequences from fish with reported genomes was retained and also with one of the two zebrafish TGF-β2, confirming our suggestion that the TGF-β that we have cloned is a novel TGF-β which is unique to fish and is different from the three known isoforms of fish TGF-β. The mature novel TGF-β is 114 aa in *S. aurata*, *Tetraodon*, *Fugu*, medaka and stickleback but differs from the length of TGF-βs of other fish species which are 112 aa long (Fig. 4).

**Table 1:** Comparison between *S. aurata *mature TGFβ and several known vertebrate TGFβs

	Amino acids	% identity
Stickleback TGF-β ENSGACG00000016971	**112/114**	**98.2**
Medaka TGF-β2 ENSORLG00000002336 (novel?)	**110/114**	**96.5**
*Tetraodon *TGF-β GSTENG10000619001	**107/114**	**93.9**
Fugu TGF-β ENSTRUG00000013167	**106/114**	**93.0**
Zebrafish TGF-β2 XM_683088 (novel?)	**99/114**	**86.8**
Zebrafish TGF-β2 NM_194385	79/114	69.3
Human TGF-β2	77/114	67.5
Mouse TGF-β3	79/114	69.3
*Sparus aurata *TGF-β1	52/114	45.6
Zebrafish TGF-β3	54/114	47.4
Chicken TGF-β3	79/114	69.3
Chicken TGF-β2	77/114	67.5

**Table 2:** Pairwise alignments of *S. aurata *TGF-β6 precursor with several fish and chicken TGF-βs

Sequence	1:	Sa_TGFbeta_FJ966093	420	aa					
Sequence	2:	Tn_HP_TGFbeta_GSTENG10000619001	420	aa	Sequences	( 1:2 )	Aligned.	Score:	**89**
Sequence	3:	Ol_HP_TGFbeta2_ENSORLG00000002336	420	aa	Sequences	( 1:3 )	Aligned.	Score:	**89**
Sequence	4:	Tr_HP_TGFbeta_ENSTRUG00000013167	420	aa	Sequences	( 1:4 )	Aligned.	Score:	**90**
Sequence	5:	Sa_TGFbeta1_AAN03842	379	aa	Sequences	( 1:5 )	Aligned.	Score:	33
Sequence	6:	Ga_HP_TGFbeta_ENSGACG00000016971	419	aa	Sequences	( 1:6 )	Aligned.	Score:	**92**
Sequence	7:	Dr_predicted_TGFbeta2_XP688180	414	aa	Sequences	( 1:7 )	Aligned.	Score:	**74**
Sequence	8:	Dr_TGFbeta2_NM194385	411	aa	Sequences	( 1:8 )	Aligned.	Score:	51
Sequence	9:	Dr_TGFbeta3_AAQ18013	410	aa	Sequences	( 1:9 )	Aligned.	Score:	46
Sequence	10:	Gg_TGFbeta3_NP990785	412	aa	Sequences	( 1:10 )	Aligned.	Score:	47
Sequence	11:	Gg_TGFbeta2_NP001026216	412	aa	Sequences	( 1:11 )	Aligned.	Score:	54

**Figure 5:**
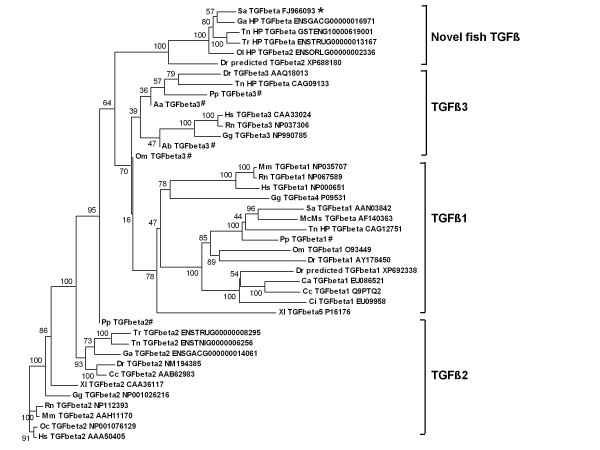
**Phylogenetic tree obtained by amino acid sequence comparison of different TGF-βs**. The phylogenetic tree was constructed from a single multiple alignment, using neighbor-joining method with Clustal X 2.0.8 and MEGA 4.0 tools. Numbers at the tree nodes represent percentage bootstrap values after 1000 replicates. *denotes the cloned TGF-β from *S. aurata *in the current study. For each sequence, NCBI or Ensembl accession number and species abbreviation are shown. Few fish species with only partial sequences available were included as well and are denoted by #. Full scientific names of species abbreviations, common names and their respective accession numbers are detailed in Additional file 2.

In order to substantiate our interpretation, an extensive CLUSTAL analysis was performed using all fish TGF-β sequences known to date and many other vertebrate TGF-βs. The phylogenetic tree that was derived clearly demonstrates that the six fish TGF-βs are clustered together. Fish and other vertebrate TGF-β1, TGF-β2 and TGF-β3 formed three additional separate clusters (Fig. 5). Full names and accession numbers used in this analysis are detailed in Additional file 2.

**Figure 6:**
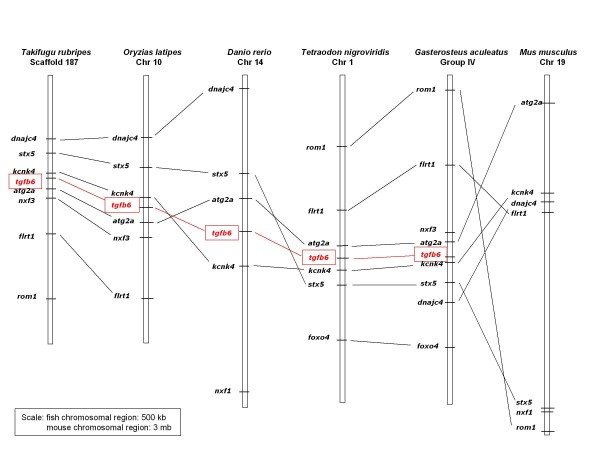
**Conserved synteny around TGF-β6 locus in teleost fish**. Syntenic relationships of genes in the vicinity of TGFβ6 gene was established using Ensembl informations [49]. Graphical view of syntenic relationships in zebrafish, medaka, stickleback, *Tetraodon *and *Fugu *TGF-β6 locus vicinity. A corresponding region in mouse is included to demonstrate conserved synteny but absence of TGF-β6 and uniqueness of this gene to teleosts.

**Figure 7:**
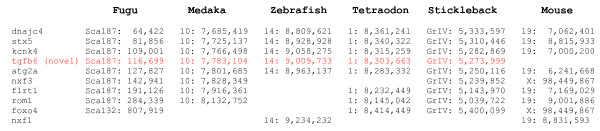
**Location of markers used in the synteny analysis on the corresponding numbered chromosome or scaffold**.

Synteny analysis of the novel TGF-β6 in five teleost genomes available to date revealed a conserved synteny around this locus. A graphic view of the syntenic relationships in zebrafish, medaka, stickleback, *Teraodon *and *Fugu*, is shown in Fig. 6 and the chromosomal location of the different markers is shown in Fig. 7. No orthologues were identified in tetrapode genomes, confirming our conclusion that this is a novel fish TGF-β. However, few of the markers used in our synteny analysis were identified on mouse Chr 19 but no TGF-β was found. The mouse Chr 19 data is included in Fig. 6 (although the scale is 3 mb in mouse compared to 500 kb in fish). TGF-β1 is on mouse Chr 1, TGF-β2 on Chr 7 and TGF-β3 on Chr 12.

**Figure 8:**
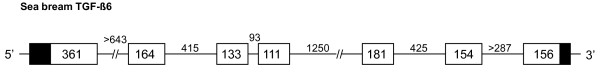
**Genomic organization and exon size of *S. aurata *TGF-β6**. Empty boxes represent exons, black boxes represent untranslated regions and lines between represent introns. Size of coding exons and of introns are indicated in bp.

During cloning of the amino-terminus of *S. aurata *TGF-β, several genomic fragments were obtained by genome-walking strategy. This approach has proven to be successful primarily due to the relatively short introns. Exon-intron boundaries of *S. aurata *TGF-β6 were identified by sequence analysis of the PCR amplified genomic DNA fragments and a comparison with known TGF-β sequences using BLAST and CLUSTAL programs. Our results revealed that *S. aurata *TGF-β6 gene is longer than 4 kb and consists of seven exons of 361, 164, 133, 111, 181, 154, and 156 bp in length. The exons are separated by six introns of >643, 415, 93, 1250, 425 and >287 bp in length. The gene structure is illustrated schematically in Fig. 8. Exon 1 forms the largest exon, whereas exon 4 is the smallest. The 23 amino acid signal peptide and 97 amino acids of the predicted propeptide are encoded by exon 1. The remaining 186 amino acids of the predicted propeptide are encoded by exons 2 to 5. The last 11 amino acids of exon 5 together with exons 6 and 7, code for the predicted mature peptide (see Fig. 3).

The gene organization in terms of exon length is identical to that of *Tetraodon*, *Fugu *and medaka, followed by zebrafish and stickleback. Among other vertebrates, the gene structure is most similar to that of mouse and chicken TGF-β2 genes (Table 3).

**Table 3:** Comparison of TGF-β exon sizes (bp) within the coding regions of vertebrate TGF-β genes

Species	Isoform	Exon1	Exon2	Exon3	Exon4	Exon5	Exon6	Exon7
***S. aurata***	**TGF-β6**	**361**	**164**	**133**	**111**	**181**	**154**	**156**
*S. aurata*	TGF-β1	595	235	151	111	48	-	-
*O. mykiss*	TGF-β	340	270	78	151	151	111	45
*D. rerio*	TGF-β3	346	164	130	108	172	154	156
***D. rerio***	**predictedTGF-β2**	349	**164**	**133**	**111**	178	151	**156**
***G. aculeatus***	**predictedTGF-β**	355	**164**	136	**111**	**181**	**154**	**156**
*T. rubripes*	predictedTGF-β2	364	164	133	111	175	154	156
***T. rubripes***	**predictedTGF-β**	**361**	**164**	**133**	**111**	**181**	**154**	**156**
***O. latipes***	**predictedTGF-β2**	**361**	**164**	**133**	**111**	**181**	**154**	**156**
***T. nigroviridis***	**predictedTGF-β**	**361**	**164**	**133**	**111**	**181**	**154**	**156**
*H. Sapiens*	TGF-β1	355	161	118	78	148	154	156
*M. musculus*	TGF-β2	346	164	133	111	178	154	156
*H. Sapiens*	TGF-β3	352	164	130	108	172	154	156
*G. gallus*	TGF-β2	343	164	133	111	175	154	156
*G. gallus*	TGF-β3	352	164	130	108	172	154	156
*X. laevis*	TGF-β5	334	158	118	75	151	154	156

Accession Nos (or gene ID from Ensembl): *S. aurata *TGF-β6, FJ966093; *S. aurata *TGF-β1, AF510084; *O. mykiss *TGF-β, AJ007836; *D. rerio *TGF-β3, AY744922 to AY744929; *D. rerio *predictedTGF-β2, LOC559723; *G. aculeatus*, predictedTGF-β, ENSGACG00000016971; *T. Rubripes *predictedTGF-β2, ENSTRUG00000008295; *T. rubripes *predictedTGF-β, ENSTRUG00000013167; *O. latipes *predictedTGF-β2, ENSORLG00000002336; *T. nigroviridis *predictedTGF-β, GSTENG10000619001; *H. sapiens *TGF-β1, X05839/X05840/X05843/X05844/X05849/X05850; *H. sapiens *TGF-β3, X14149; *M. musculus *TGF-β2, NM_009367; *G. gallus *TGF-β2, X59071/X59080/X59081/X59082; *G. gallus *TGF-β3, X58127/X60091/X60090; *X. laevis *TGF-β5, AF009331/AF009332/AF009333/AF009334/AF009335.

The novel TGF β6 in the six fish species is shown in bold letters. Identical exon length of the novel fish TGF-β6 in the six fish species is also shown in bold letters.

### Tissue distribution and developmental expression of TGF-β6

**Figure 9:**
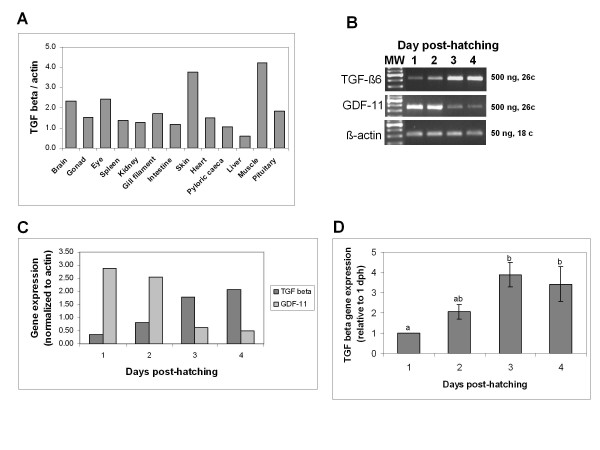
**Gene expression of TGF-β6 in different tissues and during development**. Total RNA prepared from different tissues excised from a single adult fish (A) and RNA from pools of larvae collected on 1-4 days after hatching (B, C, D) was reverse-transcribed using oligo(dT) and subjected to PCR amplification of TGF-β6. β-actin was used as a control for equal amounts used in RT-PCR. Amplified products were analyzed on 1.5-2% agarose gels and photographed under UV illumination following staining with ethidium bromide and then a densitometric analysis was performed. The expression of TGF-β6 was compared to that of another member of the TGF-β family, GDF-11. The results are presented as a ratio to that of β-actin. (C) Quantification of the expression results shown in panel (B). (D) Mean of TGF-β6 expression in three different batches of larvae collected May 1992, March 1994 and May 1994 expressed as a ratio to that of β-actin. The values are presented relative to 1 day post-hatching (dph) which is considered as 1. Different letters indicate significant difference (*P *< 0.05).

Expression pattern of TGF-β6 in different tissues was determined by semi-quantitative RT-PCR and normalized to the expression of β-actin. Densitometric analysis of TGF-β6 expression relative to β-actin in various tissues is shown in Fig. 9A. TGF-β6 was detected in all tissues studied with highest levels found in skin and muscle, followed by brain, eye and pituitary, gonad, spleen, kidney, gill filament, heart and pyloric caeca. Very low levels were detected in liver. This analysis used tissues collected from a single fish.

Ontogeny of TGF-β6 during *S. aurata *development revealed presence of transcript as early as day one after hatching. The levels of expression increased as development progressed. A representative gel of the first 4 days is shown in Fig 9B. TGF-β6 transcripts could not be detected during embryonic stages (data not shown). As a control, expression of another member of the TGF-β family (GDF-11) is included, which is high on early days post-hatching and decreases progressively with age. The levels of β-actin expression are included as well. Densitometric analysis of TGF-β6 and GDF-11 expression, relative to β-actin, during early post-hatching period, is shown in Fig. 9C. TGF-β6 and GDF-11 displayed an inverse pattern of expression: while TGF-β6 increased gradually, that of GDF-11 was high and decreased gradually. The pattern of TGF-β6 expression during early post-hatching period was verified by using four different preparations of RNA from pools of larvae. The mean of these four determinations is shown in Fig. 9D.

### Effect of food deprivation on TGF-β6 and MSTN expression in *S. aurata *skeletal white muscle

**Figure 10:**
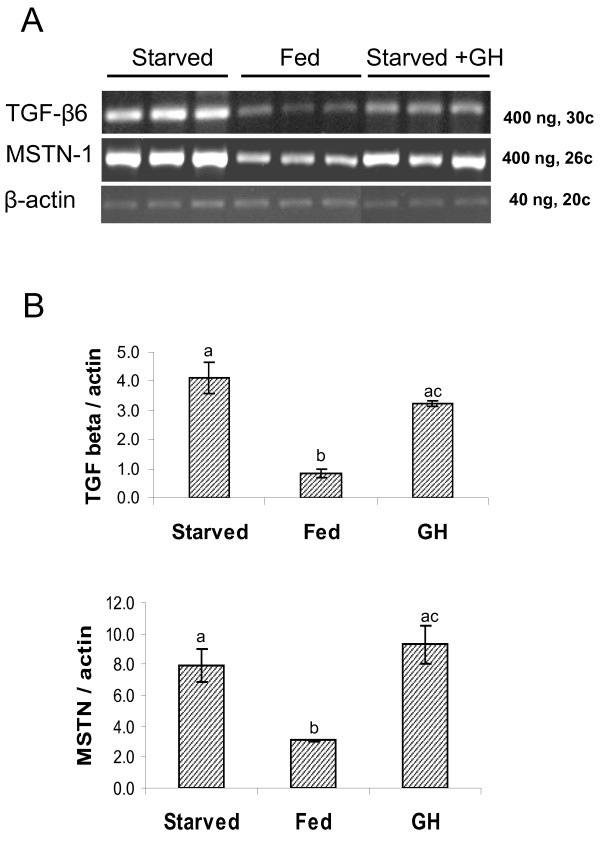
**Effect of starvation on TGF-β6 gene expression in white muscle**. Juvenile fish were starved for 5 days and then injected with PBS or GH (1 μg/gr body weight) or fed 2% of their body weight. All fish were sacrificed six hours after injections. Total RNA from white muscle of experimental fish was reverse-transcribed using oligo(dT) and subjected to PCR amplification of TGF-β6. β-actin was used as a control for equal amounts used in RT-PCR. Amplified products were analyzed on 1.5-2% agarose gels (A) and photographed under UV illumination following staining with ethidium bromide and then a densitometric analysis was performed (B). The expression of TGF-β6 was compared to that of another member of the TGF-β family, MSTN that is involved in muscle growth. The results are presented as a ratio to that of β-actin. Different letters indicates significant difference (*P *< 0.05).

Starvation of juvenile fish for 5 days resulted in 5-fold increase in TGF-β6 transcript levels in skeletal white muscle, compared to that in control fed fish. A similar effect of food deprivation was observed with respect to MSTN transcript levels, although the increase was only 2.6-fold. Injection of recombinant GH to fasted fish had minor effects on this elevation, when gene expression levels were determined 5 hours after injection (Fig. 10A, B).

## Discussion

In the present study, we have cloned and characterized cDNA and gene encoding a novel TGF-β from the marine fish *S. aurata*, which we named TGF-β6. The gene encodes a preproTGF-β of 420 amino acid long precursor. The signal sequence for secretion is predicted to be 23 residues; the prodomain is predicted to be 283 aa long and the carboxy-terminal mature TGF-β is 114 amino acid residues long. This novel TGF-β was identified in genomes of five fish species: *Tetraodon*, *Fugu*, medaka, stickleback and zebrafish, and in all but zebrafish the mature peptide is 114 residues long. Most mature TGF-βs have 112 residues, with the exception of chicken TGF-β4 mature peptide that contains 114 amino acids [10]. The 114 amino acids include nine conserved cysteines, eight of which are known to form four intrachain disulfide bonds and the ninth cysteine forming an interchain disulfide bond, resulting in a TGF-β dimer [51,52].

The precursor TGF-β6 contains three potential N-linked glycosylation sites (N-X-T/S), similar to human TGF-β1 and TGF-β2 [53] but differing from *S. aurata *TGF-β1 that has four potential N-linked glycosylation sites [22]. No RGD integrin binding site was found in the novel *S. aurata *TGF-β, in contrast to several other TGF-βs, including that of *S. aurata *TGF-β1 [22]. This site is also absent in human TGF-β2 but is present in both human TGF-β1 and TGF-β3 precursors [53]. Computer prediction suggests that the cleavage site between the leader sequence and the prodomain is between Gly^23 ^and Iso^24^, immediately in front of the INTC motif, in accordance with other TGF-β sequences in which the cleavage site is typically predicted to be immediately in front of the LSTC motif. Moreover, out of the ten residues in human TGF-β3 found to be directly involved in binding to the type II receptor: R25, K31, W32, H34, K37, Y90, Y91, V92, G93, R94 [54], eight residues are conserved in *S. aurata *mature TGF-β6.

The novel *S. aurata *TGF-β6 gene is organized in seven exons and six introns, in accordance with several other known TGF-β genes like human TGF-β1 and TGF-β3, chicken TGF-β2 and TGF-β3, zebrafish TGF-β3 and *Xenopus *TGF-β5 [27,53,55-58]. An exception is TGF-β1 from *S. aurata *which consists of 5 exons only [22]. So far, only four teleost TGF-β genes have been characterized (including the current study), although during our synteny analysis we identified the novel gene in genomes of *Tetraodon*, *Fugu*, medaka, stickleback and zebrafish. The new *S. aurata *TGF-β gene is more than 4.4 kb long (the exact size could not be determined since the first and last introns were not sequenced in full). The overall gene organization of TGF-β6 in terms of exon length is conserved in all six novel fish genes and also similar to mouse and chicken TGF-β2 genes [56], but differs substantially from two of the fish genes reported: *S. aurata *TGF-β1 (with five exons) and rainbow trout TGF-β [16,22]. In contrast, the organization of *S. aurata *TGF-β6 is similar (but not identical) to that of zebrafish TGF-β3 [27]. The most conserved exons among vertebrates including zebrafish TGF-β3 and *S. aurata *TGF-β6 are exon 6 (154 bp) and exon 7 (156 bp). The two other fish TGF-β genes that were reported appear to be organized in a completely different way: *S. aurata *TGF-β1 has five exons and rainbow trout TGF-β gene has seven exons, but lacks vertebrate intron 2, resulting in exon 2 comprising of vertebrate exon 2 and 3. It has instead a sixth new intron splitting the seventh exon into two shorter exons, with a similar length to that of *S. aurata *TGF-β1 [16,22]. In light of our results regarding *S. aurata *TGF-β6 gene organization and that of zebrafish TGF-β3 [27] as well as the five novel fish TGF-β6 genes from the database, it seems that the conclusion that intron 2 of human, chicken and *Xenopus *TGF-β isoforms is not present in the teleost TGF-β [22] is true only in these two TGF-β1 genes. Additional fish TGF-β genes need to be cloned in order to resolve this important issue. Interestingly, zebrafish predicted-TGF-β2 gene has very long introns 1, 4, 5 and 6 (range 2,500-8,900 bp), while introns 2 and 3 are short (less than 200 bp). Zebrafish TGF-β3 gene also has very long introns [27]. This contradicts an incorrect generalized statement put forward that introns in lower vertebrates are much smaller in size [22].

An extensive BLAST and CLUSTAL analyses and the resulting phylogenetic tree that was generated showed that the novel *S. aurata *TGF-β precursor is clustered together with a zebrafish TGF-β identified mistakenly as TGF-β2 (accession no. XP688180) and four additional fish genes identified through synteny analysis: *Tetraodon, Fugu*, medaka and stickleback. Other vertebrate TGF-βs were clustered into main three groups: TGF-β1, TGF-β2, and a cluster of TGF-β3. The synteny analysis indicated a high conservation in the region of the novel gene between the five fish genomes. The markers used in this synteny analysis were found also in mouse but no TGF-β was found in this region. Taken together, these results led us to the conclusion that the TGF-β cloned in the current study represents a novel TGF-β, unique to fish which we named it TGF-β6. The percent identities of the mature new peptide with mammalian and chicken TGF-β2 and TGF-β3 were in the range of 67.5 to 69.3% while that with the novel zebrafish TGF-β was 86.8% and with the four other novel genes 93-98%. Pairwise alignments of the novel *S*. *aurata *TGF-β precursor scored 89-92 with the *Tetraodon, Fugu*, medaka and stickleback predicted TGF-β and 74 with novel zebrafish TGF-β while scores of 33, 46 and 51 were obtained for pairwise alignments with *S. aurata *TGF-β1, zebrafish TGF-β2 and zebrafsih TGF-β3, respectively. These comparisons reinforce our conclusion that we have identified a new TGF-β isoform which is not one of the already characterized fish TGF-β isoforms.

Human genes for TGF-β1, -β2 and -β3 map to chromosmes 19q3.1-13.3, 1q41 and 14q23-24, respectively [59-61], and to chromosomes 7, 1 and 12 in the mouse [60,62,63]. Chicken TGF-β3 is physically located on chromosome 5 [57]. As discussed above, the zebrafish TGF-β gene homologous to the novel *S. aurata *TGF-β gene is localized on chromosome 14 and that of *Tetraodon *homologous gene on chromosome 1. The chromosomal localization of the TGF-β genes suggests that they have become widely dispersed during their evolution. It had been suggested [64] that the TGF-β family has evolved from a series of gene duplications and the genes became separated through chromosomal translocations.

The expression studies in the current report used a set of primers that span introns 5 and 6, thus ensuring that the amplified fragment by RT-PCR is the result of RNA amplification and not genomic amplification. Expression was found in all tissues studied with various levels of expression. The broad tissue distribution of TGF-β6 shown here suggests that it may have a role in a variety of tissues, and further studies should be carried out in order to shed light on possible roles for TGF-β6 in various tissues in fish. Nevertheless, this wide pattern of distribution is consistent with a wide expression reported for TGF-β1 in rainbow trout [17], *S. aurata *[22] and grass carp [37]. It is noteworthy, however, that whereas no expression was seen for TGF-β1 in *S. aurata *and grass carp liver, consistent with our current study showing very low expression of TGF-β6 in liver, rainbow trout liver expressed TGF-β1. The semi-quantitative assay in grass carp showed high expression of TGF-β1 in immune system-related organs: thymus, head kidney and spleen [37].

Skeletal muscle is a dynamic tissue that demonstrates great plasticity in response to environmental and hormonal factors. Recent studies indicated that contractile activity, nutrients, growth factors, and cytokines all contribute to determining muscle mass. Muscle responds not only to endocrine hormones but also to the autocrine production of growth factors and cytokines. Skeletal muscle synthesizes anabolic growth factors such as IGF-I and inhibitory cytokines such as TGF-βs and MSTN. These self-regulating inputs in turn influence muscle metabolism, including the use of nutrients such as glucose and amino acids.

The natural life cycle of many fish species includes seasonal cycles of low temperature coupled with restricted food supply in the winter leading to reduced protein synthesis and slower growth. Although numerous laboratory experiments in fish tested the effects of food deprivation (starvation) and re-feeding on the expression of the GH-IGF system and several muscle regulatory factors [65-70], this is the first report on a possible involvement of TGF-β in fish muscle growth as well as in the effect of food deprivation. Following a 5-day period of starvation, steady state levels of transcripts for both TGF-β6 and MSTN increased in skeletal muscle, compared to fish fed normally. The immediate cause for this increase in transcript levels in fish skeletal muscle in response to starvation is not clear and more studies should be done in the future. Nevertheless, a similar effect of increased expression levels of MSTN in skeletal muscle was reported after a prolonged starvation of sea bass [69] and underfeeding in sheep [71]. Since the IGF axis is nutritionally sensitive *in vivo *and since IGFs, TGF-βs and MSTN are important regulators of vertebrate muscle growth, we can speculate that also in the starved fish in the current study an interaction exists between the IGF axis and TGF-β/MSTN, resulting in elevated expression of TGF-β6 and MSTN in skeletal muscle. One way for such an interaction might be a cross-talk between the mediators or regulators of IGF-I and MSTN, as IGF-I induces expression or activity of myogenic regulatory factors like MyoD and myogenin [72] and MSTN is a downstream target of MyoD [73]. Another explanation might be the known effect of starvation on lowering circulatory IGF-I and GH, resulting in removing a possible inhibitory effect known to be exerted by GH on skeletal MSTN levels *in vivo *and *in vitro *[74]. Moreover, IGFs suppressed expression of the myogenesis-inhibiting TGF-β *in vitro *[75]. Information on TGF-β expression during fish ontogeny is available to date only for TGF-β3 in the laboratory model, the zebrafish [27], but not for other fish species or other TGF-β isoforms. In the zebrafish, TGF-β3 was expressed at extremely low levels in embryos from 64-cell (2 hpf) stage up to the 10-somite (14 hpf) stage, with significant higher expression observed only in the 18-somite (18 hpf). These results differ from our current study, in which we could not detect TGF-β6 transcripts earlier than day of hatching and then the levels were very low. A continuous increase in expression was seen as development progressed. This observation is not due to technical problems, since high levels of GDF-11, a TGF-β superfamily member, were found in *S. aurata *embryos aged 15-16 h and 30 h (data not shown) and the levels of GDF-11 expression decreased as development progressed from day 1 to day 4 post-hatching. For comparison with other vertebrates, in chick embryos very low expression of TGF-β2 was reported during first 6 days [76], while in the rat TGF-β2 expression was high in skeletal muscle of embryonic day 14, decreased on postnatal day 3 and was negligible in adult rat. A similar pattern of decrease was observed for TGF-β2 peptide in skeletal muscle during postnatal development [32,77].

## Conclusion

A novel TGF-β isoform was cloned and characterized from *S. aurata *and named TGF-β6. The deduced amino acid of the precursor showed very high homology with five entries in the database for *Tetraodon, Fugu*, medaka, stickleback and zebrafish TGF-βs, but less identity with other known vertebrate TGF-βs. The gene organization is conserved among these fish species but also similar to mouse and chicken TGF-β2 genes. Putative functional sites have been recognized in the translated peptide and in the nucleotide sequence. TGF-β6 transcripts are ubiquitously expressed with high expression seen in skeletal muscle. It is developmentally regulated and is also affected by nutritional state. The importance of TGF-β6 for skeletal muscle growth in fish needs to be further investigated, and in particular, its response to food deprivation.

## Authors' contributions

BF and SBJ conceived and initiated the project. EO participated in the cloning part under the supervision of BF. Analysis of sequences and expression studies were done by BF. All authors read and approved the final manuscript.

## Supplementary Material

Additional file 1 **Primers used for cloning and expression of *S. aurata *TGF-β6**. Primer names, sequences and annealing temperatures used for cloning and for expression of *S. aurata *TGF-β6 Click here for file

Additional file 2 **Full species names and accession numbers of TGF-β isoforms**. List of isoforms, species common names, abbreviations, species scientific names and Genbank accession numbers used in alignments and phylogenetic analyses. Click here for file
