# Supplementary material for: Identification of a novel transforming growth factor-β (TGF-β6) gene in fish: regulation in skeletal muscle by nutritional state
Source: BMC Mol Biol. 2010 May 12;11:37. doi: 10.1186/1471-2199-11-37 (PMC2881917; doi:10.1186/1471-2199-11-37)
Supplement: Additional file 1 — Primers used for cloning and expression of S. aurata TGF-β6. Primer names, sequences and annealing temperatures used for cloning and for expression of S. aurata TGF-β6 [file 1471-2199-11-37-S1.DOC]

**Additional file 1. Primer names, sequences and annealing temperatures used for cloning and for expression of *S. aurata* TGF-β6**

| **Annealing**  **Temp [ºC]** | **Use** | **Sequence** | **Primer** |
| --- | --- | --- | --- |
| 54-62 | PCR | 5’-TGGAA(A/G)TGGGT(C/G)CA(T/C)GA(G/A)C-3’  A A C C G | TGF-1  *Sparus* sequence*** |
| 46-56 | PCR | 5’-CAT(A/G)TT(G/T)GA(G/A/C)AG(C/T)TG(C/T)TC-3’  G G G T C | TGF-2  *Sparus* sequence*** |
| 50 | genomic | 5’-GTTGGCTTTGTAACCTTT-3’ | TGF-5 |
| 64 | 3’RACE | 5’-GGCCGCACACCTCGCGTC-3’ | TGF-7 |
| 62 | 3’RACE | 5’-TGTGCCGGGAGCTGTCCG-3’ | TGF-8 |
| 62 | genomic | 5’-CGGACAGCTCCCGGCACA-3’ | TGF-8R |
| 58 | genomic | 5’-AATCGGCCTGACCTTTGAC-3’ | TGF-9 |
| 64 | genomic | 5’-TAGCTTCTCGTCATCCACACC-3’ | TGF-10 |
| 62 | gene expression | 5’-GTGGATGACGAGAAGCTACG-3’ | TGF-11 |
| 66 | gene expression | 5’-GCGGCACTTGCAGGATTTGAC-3’ | TGF-12 |
| 62 | genomic | 5’-CACAGGGACCAGGGTCTAAA-3’ | TGF-13 |
| 60 | genomic | 5’-CTGACAATGAAGTCCAGCGA-3’ | TGF-14 |
| 62 | genomic & 5’RACE | 5’-GTGCTCTCTTCGTCTGGCTT-3’ | TGF-15 |
| 64 | 5’RACE | 5’-CGAAGTGGACGACCCTGTAG-3’ | TGF-16 |
| - | genomic | 5’-GTTCATCTTTACAAGCTAGCGCTGAACAATGCTGTGGACAAGCTTGAATTC-3’ | Oligo1 |
| - | genomic | 5’-GTTCGAACTTAAG-3’ | Oligo2 |
| 60 | genomic | 5’-GTTCATCTTTACAAGCTAGCG-3’ | L1 |
| 54 | genomic | 5’-TCCTGAACAATGCTGTGG-3’ | L2 |
| 72 | 5’RACE | 5’-GCTGATGGCGATGAATGAACACTG-3’ | 5’RACE outer |
| 110 | 5’RACE | 5’-CGCGGATCCGAACACTGCGTTTGCTGGCTTTGATG-3’ | 5’RACE inner |
| 66 | 3’RACE | 5’-GCGAGCACAGAATTAATACGACT-3’ | 3’RACE outer |
| 94 | 3’RACE | 5’-CGCGGATCCGAATTAATACGACTCACTATAGG-3’ | 3’RACE inner |
| - | RT | 5’-GCGAGCACAGAATTAATACGACTCACTATAGG(T)_12_VN-3’ | 3’RACE adapter |
| - | 5’RACE | 5’-GCUGAUGGCGAUGAAUGAACACUGCGUUUGCUGGCUUUGAUGAAA-3’ | 5’RACE adaptor |
| 54 | 3’RACE | 5’-GACTCGAGTCGACATCG | Adaptor |
| - | 3’RACE | 5’-GACTCGAGTCGACATCGA(T)_17_-3’ | dT-adaptor |
| 92 | gene expression | 5’-CGCGCATATGGACTCGGGCCTGGACTGT-3’ | MSTN-3 |
| 100 | gene expression | 5’-GCGCGGATCCTCAAGAGCATCCACAACGGTC-3’ | MSTN-4 |
| 60 | gene expression | 5’-CTTCAGCACCTTTGTGAACAT-3’ | R-GDF-1 |
| 88 | gene expression | 5’-CGCGCATATGGATGAGCCCAATCTGCTG | GDF-4 |
| 64 | gene expression | 5’-CGACGGACAGGTCATCACCA-3’ | Act-1 |
| 62 | gene expression | 5’-AGAAGCATTTGCGGTGGACG-3’ | Act-2 |

*Nucleotide changes found in saTGFß-6 cDNA compared to the primers used for cloning
