# Supplementary material for: Identification of a novel transforming growth factor-β (TGF-β6) gene in fish: regulation in skeletal muscle by nutritional state
Source: BMC Mol Biol. 2010 May 12;11:37. doi: 10.1186/1471-2199-11-37 (PMC2881917; doi:10.1186/1471-2199-11-37)
Supplement: Additional file 2 — Full species names and accession numbers of TGF-β isoforms. List of isoforms, species common names, abbreviations, species scientific names and Genbank accession numbers used in alignments and phylogenetic analyses. [file 1471-2199-11-37-S2.DOC]

**Additional file 2. List of isoforms, species common names, abbreviations, species scientific names and Genbank accession numbers used in alignments and phylogenetic analyses**

________________________________________________________________________________________________________

*Isoform*  *Species* *Abbreviation* *Common name* *Genebank accession no*

*____________________________________________________________________________ * from Ensembl genome browser 56*

TGF-ß6 *Sparus aurata* Sa Gilthead sea bream FJ966093

TGF-ß1 *Sparus aurata* Sa Gilthead sea bream AF424703, AAN03842 (aa)

TGF-ß1 *Oncorhynchus mykiss* Om Rainbow trout AJ007836, O93449 (aa)

TGF-ß3 *Oncorhynchus mykiss* Om Rainbow trout Laing et al., 1999

HP TGF-ß1? *Tetraodon nigroviridis* Tn Green spotted puffer CAG12751 (aa)

HP TGF-ß3? *Tetraodon nigroviridis* Tn Green spotted puffer CAG09133 (aa)

TGF-ß2 *Tetraodon nigroviridis* Tn Green spotted puffer ENSTNIG0000006256*

HP TGF-ß *Tetraodon nigroviridis* Tn Green spotted puffer GSTENG10000619001*

TGF-ß1 *Danio rerio* Dr Zebrafish AY178450

PredictedTGF-ß1 *Danio rerio* Dr Zebrafish XM_687246, XP_692338 (aa)

TGF-ß2 *Danio rerio* Dr Zebrafish NM_194385

PredictedTGF-ß2 *Danio rerio* Dr Zebrafish XM_683088, XP_688180 (aa)

TGF-ß3 *Danio rerio* Dr Zebrafish AAQ18013 (aa)

HP TGF-ß2 *Oryzias latipes* Ol Medaka ENSORLG0000002336*

HP TGF-ß2 *Takifugu rubripes* Tr Japanese puffer fish ENSTRUG00000008295*

HP TGF-ß *Takifugu rubripes* Tr Japanese puffer fish ENSTRUG00000013167*

HP TGF-ß *Gasterosteus aculeatus* Ga Three-spined stickleback ENSGACG00000016971*

TGF-ß2 *Gasterosteus aculeatus* Ga Three-spined stickleback ENSGACG000000014061*

TGF-ß *Morone chrysops x Morone saxatilis* McMS Hybrid sriped bass AF140363

TGF-ß1 *Cyprinus carpio* Cc Common carp AF136947, Q9PTQ2 (aa)

TGF-ß2 *Cyprinus carpio* Cc Common carp U66874, AAB62983 (aa)

TGF-ß1 *Ctenopharyngodon idella* Ci Grass carp EU099588

TGF-ß1 *Carassius auratus* Ca Goldfish EU086521

TGF-ß3 *Acipenser baeri* Ab Siberian sturgeon Laing et al., 1999

TGF-ß3 *Angilla angilla* Aa European eel Laing et al., 1999

TGF-ß1 *Pleuronectes platessa* Pp Plaice Laing et al., 2000

TGF-ß2 *Pleuronectes platessa* Pp Plaice Laing et al., 2000

TGF-ß3 *Pleuronectes platessa* Pp Plaice Laing et al., 2000

TGF-ß5 *Xenopus laevis* Xl African clawed frog P16176 (aa)

TGF-ß2 *Xenopus laevis* Xl African clawed frog CAA36117 (aa)

TGF-ß4 *Gallus gallus* Gg Chicken (white leghorn) P09531 (aa)

TGF-ß2 *Gallus gallus* Gg Chicken NM_001031045, NP_001026216 (aa)

TGF-ß3 *Gallus gallus* Gg Chicken NP_990785 (aa)

TGF-ß1 *Mus musculus* Mm Mouse AJ009862, NP_035707 (aa)

TGF-ß2 *Mus musculus* Mm Mouse AAH11170 (aa)

TGF-ß1 *Rattus norvegicus* Rn Rat NP_067589 (aa)

TGF-ß2 *Rattus norvegicus* Rn Rat NP_112393 (aa)

TGF-ß3 *Rattus norvegicus* Rn Rat NP_037306 (aa)

TGF-ß1 *Homo sapiens*  Hs Human NP-000651 (aa)

TGF-ß2 *Homo sapiens*  Hs Human AAA50405 (aa)

TGF-ß3 *Homo sapiens*  Hs Human CAA33024 (aa)

TGF-ß2 *Oryctolagus cuniculus* Oc Rabbit NP_001076129 (aa)
